# Patterns of Relative and Quantitative Abundances of Marine Bacteria in Surface Waters of the Subtropical Northwest Pacific Ocean Estimated With High-Throughput Quantification Sequencing

**DOI:** 10.3389/fmicb.2020.599614

**Published:** 2021-01-21

**Authors:** Jie Kong, Xin Liu, Lei Wang, Hao Huang, Danyun Ou, Jiayu Guo, Edward A. Laws, Bangqin Huang

**Affiliations:** ^1^State Key Laboratory of Marine Environmental Science, Xiamen University, Xiamen, China; ^2^Fujian Provincial Key Laboratory for Coastal Ecology and Environmental Studies, College of the Environment and Ecology, Xiamen University, Xiamen, China; ^3^Third Institute of Oceanography, Ministry of Natural Resources, Xiamen, China; ^4^Department of Environmental Sciences, College of the Coast and Environment, Louisiana State University, Baton Rouge, LA, United States

**Keywords:** relative abundance, bacterial community structure, distribution patterns, ecology of marine bacteria, quantitative abundance

## Abstract

Bacteria play a pivotal role in shaping ecosystems and contributing to elemental cycling and energy flow in the oceans. However, few studies have focused on bacteria at a trans-basin scale, and studies across the subtropical Northwest Pacific Ocean (NWPO), one of the largest biomes on Earth, have been especially lacking. Although the recently developed high-throughput quantitative sequencing methodology can simultaneously provide information on relative abundance, quantitative abundance, and taxonomic affiliations, it has not been thoroughly evaluated. We collected surface seawater samples for high-throughput, quantitative sequencing of 16S rRNA genes on a transect across the subtropical NWPO to elucidate the distribution of bacterial taxa, patterns of their community structure, and the factors that are potentially important regulators of that structure. We used the quantitative and relative abundances of bacterial taxa to test hypotheses related to their ecology. Total 16S rRNA gene copies ranged from 1.86 × 10^8^ to 1.14 × 10^9^ copies L^−1^. Bacterial communities were distributed in distinct geographical patterns with spatially adjacent stations clustered together. Spatial considerations may be more important determinants of bacterial community structures than measured environmental variables. The quantitative and relative abundances of bacterial communities exhibited similar distribution patterns and potentially important determinants at the whole-community level, but inner-community connections and correlations with variables differed at subgroup levels. This study advanced understanding of the community structure and distribution patterns of marine bacteria as well as some potentially important determinants thereof in a subtropical oligotrophic ocean system. Results highlighted the importance of considering both the quantitative and relative abundances of members of marine bacterial communities.

## Introduction

Bacteria play a pivotal role in shaping ecosystems and contributing to the cycling of elements and the flow of energy in the oceans (Ducklow, 2000; Kirchman, 2016; Steinberg and Landry, 2017). Knowledge of the distribution patterns of marine bacteria, their community composition, and the factors that shape that composition in surface seawater is of great interest, especially because of the ongoing climate changes that are expected to cause warming and acidification of the surface waters of the ocean, shoaling of the surface mixed layer, and increased irradiance within that shallower mixed layer (Hutchins and Fu, 2017). With well-developed, high-throughput sequencing (HTS) technology, it is now technologically and economically feasible to finely resolve bacterial community composition. Sequences obtained from HTS can provide both genetic (taxonomic) information and relative abundance. This information has dramatically expanded our understanding of the composition and biogeography of bacterial communities and the mechanisms by which they are assembled (Sunagawa et al., 2015; Goodwin et al., 2016; Lindh et al., 2018). Even though HTS can provide only two basic attributes of a community on relative abundance and taxonomic affiliation; it cannot provide information on absolute abundance, which is needed for an accurate and comprehensive interpretation of the biological and ecological implications of bacterial community structure (Props et al., 2017; Vandeputte et al., 2017; Zhang et al., 2017; Yang et al., 2018). The addition of artificial standard spike-ins (i.e., internal standard sequences) before HTS (that is, high-throughput quantification sequencing, HTQS) makes it possible to estimate microbial abundances (Tourlousse et al., 2017; Yang et al., 2018). Although the effects of PCR bias cannot be ruled out in HTQS and would impact estimates of microbial abundances, it has been shown that estimates of microbial community structure and composition are not perturbed by artificial standard spike-ins, and even complex 16S rRNA gene pools do not affect quantification based on artificial standard spike-ins (Tourlousse et al., 2017). In this study, we considered that gene copy numbers estimated from HTQS were metrics of quantitative abundance rather than relative abundance and could provide, at least to some extent, information about all three community attributes (i.e., taxa, relative abundance, and quantitative abundance) and could thereby enhance the significance of HTS-based microbiome studies (Tourlousse et al., 2017; Wang et al., 2018; Yang et al., 2018; Jiang et al., 2019; Mou et al., 2020). However, to our knowledge, only one study has previously applied this improved HTQS method to an aquatic ecosystem. That study linked net community production and microbial community composition in the western North Atlantic and revealed that the microbial community that resulted from an algal bloom was associated with a regional peak of net community production (Wang et al., 2018). Moreover, little is known about the differences and similarities of the implications of relative and quantitative abundances in marine microbial ecology.

The subtropical NWPO is one of the largest biomes on Earth and is characterized by warm, nutrient-poor, low-biomass, stratified surface waters (Spalding et al., 2012; Tseng et al., 2016). Although the subtropical NWPO plays a significant role in moderating the global climate and biogeochemical cycles, it is undersampled and not well understood in terms of its ecosystem structure and functionality (Tseng et al., 2016; Karl and Church, 2017; Kavanaugh et al., 2018).

There have previously been many studies of marine bacterial communities over different geographic ranges with high sample numbers and/or great depth coverage. However, those studies have been conducted at only a few scattered stations or within only a few subdivisions of the North Pacific Ocean, and only a small number of samples have been collected from the subtropical NWPO (Fuhrman et al., 2008; Zinger et al., 2011; Sunagawa et al., 2015; Lindh, 2017; Shulse et al., 2017; Li et al., 2018; Lindh et al., 2018). Zinger et al. (2011) identified the global patterns of bacterial distributions across the world's oceans by analyzing 509 samples, encompassing snapshot locations in the North Pacific Ocean. Sunagawa et al. (2015) have studied the structure and function of the global ocean microbiome based on samples from 68 locations, including six from the eastern North Pacific Ocean. Li et al. (2018) have estimated bacterial diversity and nitrogen utilization in the northwestern Pacific Ocean, and Shulse et al. (2017) have collected samples from the Clarion-Clipperton Zone of the Eastern North Pacific to examine the diversity and composition of the microbial communities. The distribution patterns and mechanisms of assembly of communities are affected by spatial scale (Martiny et al., 2011; Shi et al., 2018). To our knowledge, however, there has been no trans-basin study of marine bacteria in the North Pacific Ocean.

In this study, we collected surface seawater samples for HTQS of the 16S rRNA gene along a transect across the subtropical NWPO. The objectives of the study were to (1) reveal bacterial community structure, distribution patterns, and potential determinants thereof, and (2), to compare the quantitative and relative bacterial abundances as indicators of the roles of bacterial communities in subtropical NWPO surface seawater.

## Materials and Methods

### Sample Collection and Processing

**Figure 1:**
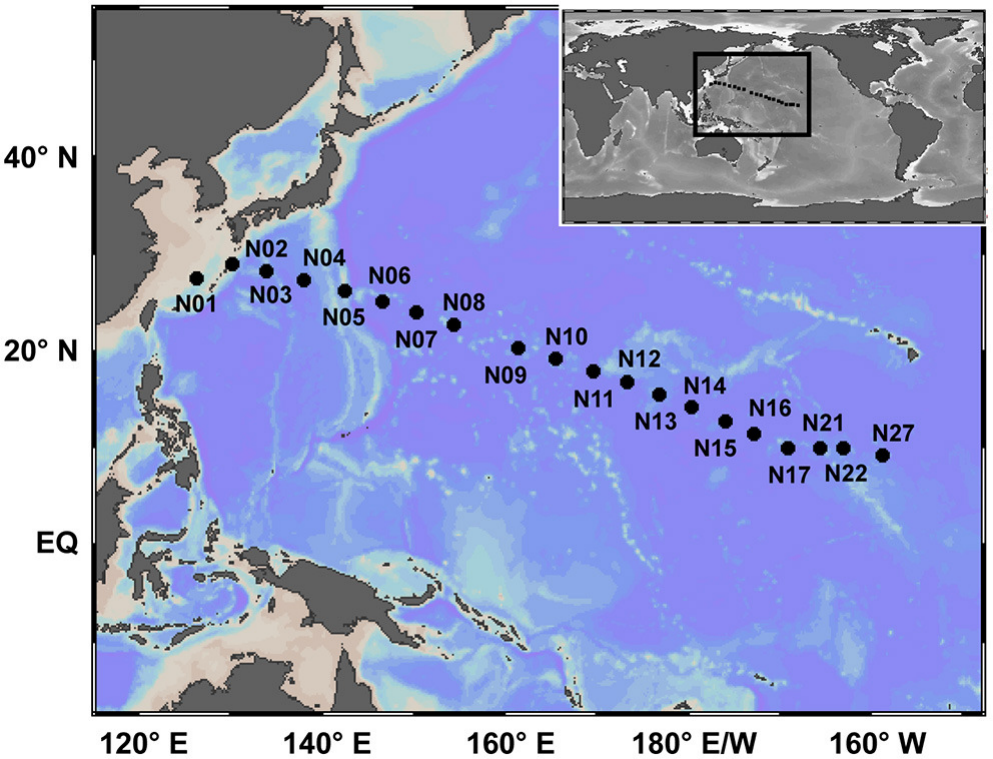
Sampling locations in the Northwest Pacific Ocean. Surface seawater samples were collected from 20 stations (marked by black dots). The map with sampling locations was constructed using Ocean Data View version 4.7.10.

Surface seawater from 20 stations located in the subtropical NWPO (Figure 1), ranging from 126.21°W to 158.77°E and from 9.31°N to 28.96°N, were collected with 12-L Niskin bottles deployed with a conductivity-temperature-depth (SBE911 plus CTD system) profiler or manually from July 16 to August 5, 2018, as a part of the Dayang 50 cruise (Supplementary Table 1). The map of sampling stations was constructed using the Ocean Data View version 4.7.10 (Schlitzer, 2015). Sea surface temperature was measured with an onboard thermosalinograph (SeaBird Electronics model SBE 45). Daily mean sea surface salinity data for each sampling location were extracted from the GLOBAL-REANALYSIS-PHY-001-30 reanalysis product (1/12° horizontal resolution) provided by the Copernicus Marine Environment Monitoring Service (http://marine.copernicus.eu/).

The seawater was passed through 200-μm nylon mesh to remove metazoans, and the cells from 4.25 to 7.3 liters of the filtrate were harvested through 47-mm-diameter polycarbonate filters (0.2-μm pore size, Millipore, USA). It was inevitable that bacteria-associated particles (> 200 μm) were also removed with the 200-μm nylon mesh. The filters were then stored at −80°C until DNA extraction. Duplicate seawater samples from each station were stored at −20°C in acid-washed, 250-mL polyethylene bottles, which were transported to the laboratory and analyzed (i) for dissolved reactive phosphorus (DRP) concentrations following the manual, solid phase extraction method described previously (Yuan et al., 2016), (ii) for NO_x_ (nitrate + nitrite) concentrations using a colorimetric method with a Flow Injection Analysis-Liquid Waveguide Capillary Cell system (Zhang, 2000) and (iii) for Si(OH)_4_ concentrations using an AA3 AutoAnalyzer (Bran+Luebbe, Germany) following procedures described previously (Du et al., 2013).

For measurement of chlorophyll *a* (Chl *a*) concentrations, 4.3 liters of seawater from each station were filtered onto Whatman 25-mm-diameter GF/F filters and stored at −80°C. Chl *a* was extracted with N, N-dimethylformamide from filters, and measured by high-performance liquid chromatography (Liu et al., 2016). Seawater samples for enumeration of bacterial abundance were fixed with 50 % paraformaldehyde (Sangon Biotech, China) at a final concentration of 1 % (v/v), incubated in the dark for 10–15 min, and stored at −80°C. Bacteria were stained with 100× SYBR Green I (final concentration 1×) (Thermo Fisher Scientific, USA) were counted using a BD Accuri C6 flow cytometer (BD Biosciences, USA) after mixing with 1-μm yellow-green latex beads (Sigma, USA) (Marie et al., 1999).

### High-Throughput Quantification Sequencing

To quantify taxa abundance across samples, HTQS, which has been described in pioneer studies (Tourlousse et al., 2017; Wang et al., 2018; Jiang et al., 2019; Mou et al., 2020), was applied and carried out mainly in Genesky Biotechnologies (China), with some modifications. Briefly, total genomic DNAs were extracted using a DNeasy PowerWater Kit (Qiagen, Germany) following the manufacturer's instructions from polycarbonate filters with concentrated cells. DNA quality and purity were evaluated by gel electrophoresis and NanoDrop 2000 (Thermo Fisher Scientific, USA), and the concentration was quantified with an Invitrogen Qubit 3.0 Spectrophotometer (Thermo Fisher Scientific, USA). Artificial standard spike-in sequences consisted of both conserved regions that were identical to natural 16S rRNA genes and artificial variable regions that were random sequences with about 40% G+C content and that shared negligible identity with sequences in the public databases. Artificial standard spike-ins were designed and synthesized in Genesky Biotechnologies (China). Nine artificial standard spike-ins were added to genomic DNAs for each sample at four different concentrations (10^3^, 10^4^, 10^5^, and 10^6^ copies of sequences for three, two, two, and two standard spike-ins, respectively), followed by amplification of the V3-V4 region of bacterial 16S rRNA gene with primers 341F and 805R in triplicate for 25 cycles (Herlemann et al., 2011). Amplicons were checked using gel electrophoresis and purified with Agencourt AMPure XP PCR Purification Beads (Beckman Coulter, USA). Purified amplicons of each sample were added with a sample-specific index sequence and then used to construct a library. The library was quantified, pooled, checked, and then sequenced using an Illumina Miseq Benchtop Sequencer (Illumina, USA) for 2 × 250 base pair (bp) paired-end reads.

### Processing and Analysis of Sequencing Data

The raw paired-end reads with Q20 values ≥97.1% and Q30 values ≥94.37% were cleaned using Trim Galore v0.4.5 (http://www.bioinformatics.babraham.ac.uk/projects/trim_galore/), FLASH2 v2.2.00 (Magoc and Salzberg, 2011), mothur v.1.39.3 (Schloss et al., 2009), and Usearch v10 (Edgar, 2013), including quality checking, filtering, and assembly of data. First, Trim Galore was applied to trim adapter and bases with quality scores <20 at the end of the read and reads shorter than 100 bp were removed. Second, FLASH2 was used to merge paired-end reads, followed by the removal of low-quality sequences (Magoc and Salzberg, 2011). Third, mothur was applied to identify and remove primers from sequences and filter out sequences with N-base or homopolymer >6 bp. Finally, sequences with total base error rates larger than two or lengths shorter than 200 were removed using Usearch, resulting in clean sequences for further processing (Edgar, 2013). UPARSE (Edgar, 2013) implemented in Usearch v10 was performed for processing chimera removal, singleton removal, Operational Taxonomic Unit (OTU) clustering (97% similarity cut-off), and picking of representative sequences.

Representative sequences were then blasted against the Silva132 database (Yilmaz et al., 2014) for taxonomic assignment. OTUs assigned to spike-in sequences were identified, counted, and removed for each sample, and the relative and quantitative abundances of the remaining OTUs were then calculated. For relative abundance analyses, an OTU table without singleton and non-bacterial sequences (i.e., spike-in, archaea, and chloroplast) was randomly rarefied to the same sequence number of 85442 (the minimum number of sequences in the samples) for each sample. For analyses of quantitative abundances, a standard curve equation based on the 9 added standard spike-ins for each sample was first constructed following *y* = *ax* + *b* (1), where y is the log-transformed number of spike-in OTU sequences, x represents log-transformed copies of the added spike-in, and the parameters a and b are fitting coefficients.

The quantitative abundance of each OTU in a sample was then determined as follows: $AB_{otu}\;=\frac{Y_{otu}}{R^{*}V}\;\left(2\right)$, where AB_otu_ is the quantitative abundance of OTUs in the unit of copies L^−1^; Y_otu_ represents OTU abundance calculated from equation (1); R is the recovery ratio of DNA concentration (sequenced genomic DNA/total genomic DNA extracted in each sample), and V represents the volume of seawater filtered. Based on relative abundance, richness (observed OTU number), Chao 1, Shannon, and Faith's phylogenetic diversity (PD) (Faith, 1992) indices were calculated by applying command alpha_diversity.py in QIIME v.1.9.0 (Caporaso et al., 2010).

### Statistical Analysis

All statistical analyses and figures were done with R (version 3.6.1) (R Core Team, 2016) unless otherwise mentioned. Spearman rank correlation tests were applied to pairs of variables, including biogeochemical factors and alpha diversity indices of bacteria, with *P*-values corrected using the false discovery rate (fdr) method. To evaluate inner-community connections, the function corr.test() (arguments: method = “spearman,” adjust = “fdr”) in package ‘psych’ (Revelle, 2017) were used to calculate the pairwise correlations for OTUs with relative abundance >0.1% (relative to total sequences), followed by depicturing significant correlations (adjusted *p* < 0.05 and *r* ≥ 0.6) and analysis of network attributes through the software Gephi (Bastian et al., 2009). Pairwise Spearman correlation coefficients between relative and quantitative abundances were analyzed for OTUs recovered in all samples with *p*-values adjusted in the package “q-value” (Dabney et al., 2010). The procrustes analysis was applied to test the agreement between relative and quantitative abundances in presenting the distribution patterns of bacterial communities using the “vegan” package (Oksanen et al., 2016).

First, Principal Coordinates Analysis was performed separately on relative and quantitative abundances of bacterial communities to reduce their dimensionalities. Then, the Procrustes analysis was used to stretch and rotate the points in principal coordinates matrices using the function procrustes(). The statistical significance was measured with a Monte Carlo test, with *M*^2^ indicating the goodness of fit. The Mantel test was applied to reveal the potential effects of factors such as environmental and geographic metrics on bacterial community composition based on Bray-Curtis distance.

To evaluate the relative effects of environmental and spatial variables in constructing the bacterial communities, a variation partitioning analysis (VPA) was performed based on redundancy analysis (RDA), as previously described (Borcard et al., 2011). A sparse partial least squares (sPLS) approach implemented in the R package “mixOmics” (Rohart et al., 2017) was performed to simultaneously select variables from bacterial subgroups (at phylum and order levels, respectively), and variables (i.e., environmental and geographic factors) that could identify certain bacterial subgroups having high correlations with specific parameters.

## Results

### Environmental Characteristics

**Figure 2:**
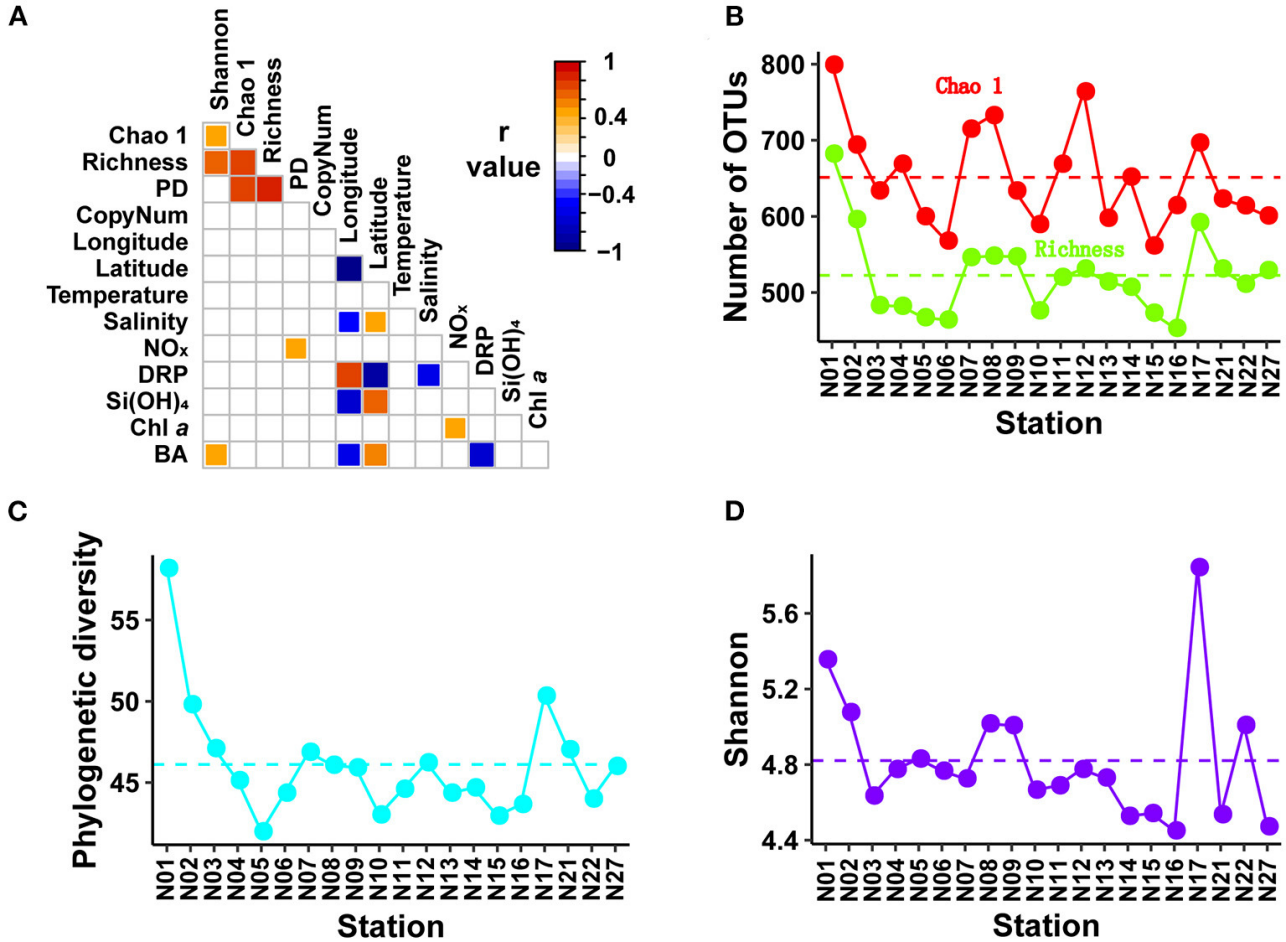
Spearman correlation matrix and distribution patterns of bacterial α-diversity indices. The Spearman correlation matrix **(A)** showing the results of Spearman rank correlation tests among α-diversity indices, environmental variables, geographic factors, and copy number of 16S rRNA gene. The *p*-values were corrected using package “psych.” Only adjusted *p* < 0.05 are shown in the Spearman correlation matrix with color indicating the *r* value. Distribution patterns of α-diversity indices including Chao1 and richness, PD, and Shannon are shown in **(B–D)**, respectively. PD, phylogenetic diversity; CopyNum, copy number of 16S rRNA gene; NO_x_, nitrite + nitrate; DRP, dissolved reactive phosphorus; BA, bacterial abundance.

Twenty surface seawater samples were collected and measured for relevant environmental variables (Supplementary Table 1, Supplementary Figure 1) across the subtropical NWPO during the summer of 2018. Sea surface temperature averaged 29.11°C (range: 28.55–29.68°C) (Supplementary Figure 1A). Sea surface salinity averaged 34.36 (range: 33.66–35.12) (Supplementary Figure 1B). Concentrations of NO_x_ (nitrate + nitrite) averaged 0.033 μmol/L (range: 0.014–0.104 μmol/L) (Supplementary Figure 1C). Dissolved reactive phosphate (DRP) concentrations averaged 0.07 μmol/L (range: 0.01–0.15 μmol/L) (Supplementary Figure 1D). The geographical distribution of Si(OH)_4_ concentrations, which averaged 1.08 μmol/L (range: 0.57–1.93 μmol/L) (Supplementary Figure 1E) was opposite that of the DRP concentrations. The Si(OH)_4_ concentrations increased from west to east across the subtropical NWPO and decreased toward the north (Supplementary Figures 1D,E, Figure 2A). The concentrations of Chl *a* averaged 25.2 ng/L (range: 5.4–48.8 ng/L) (Supplementary Figure 1F). Bacterial abundances varied from 1.15×10^5^ cells/ml to 1.68 × 10^5^ cells/ml (Supplementary Figure 1G).

### α-Diversity of Bacterial Communities

Rarefaction curves indicated that all amplicon samples were almost saturated with respect to the number of sequences. The implication of this was that the majority of bacterial communities in the amplicon samples collected from subtropical NWPO surface water were recovered at the current sequencing depth (Supplementary Figure 2). All samples rarefied to 85,442 sequences yielded a total of 1,192 OTUs at a cutoff value of 97% similarity. The samples contained 453–682 OTUs per sample (Figure 2B). The distribution patterns across the subtropical NWPO were consistent for richness based on the Chao 1, phylogenetic diversity (PD), and Shannon diversity indices. The Chao 1 ranged from 561 to 799 (average of 651), the PD index from 42 to 58 (average of 46), and the Shannon from 4.5 to 5.8 (average of 4.8). The richness, Chao 1, and phylogenetic indices peaked at station N01, whereas the maximum of Shannon index was at station N17 (Figures 2B–D).

### Structure and Distribution Patterns of Bacterial Communities

**Figure 3:**
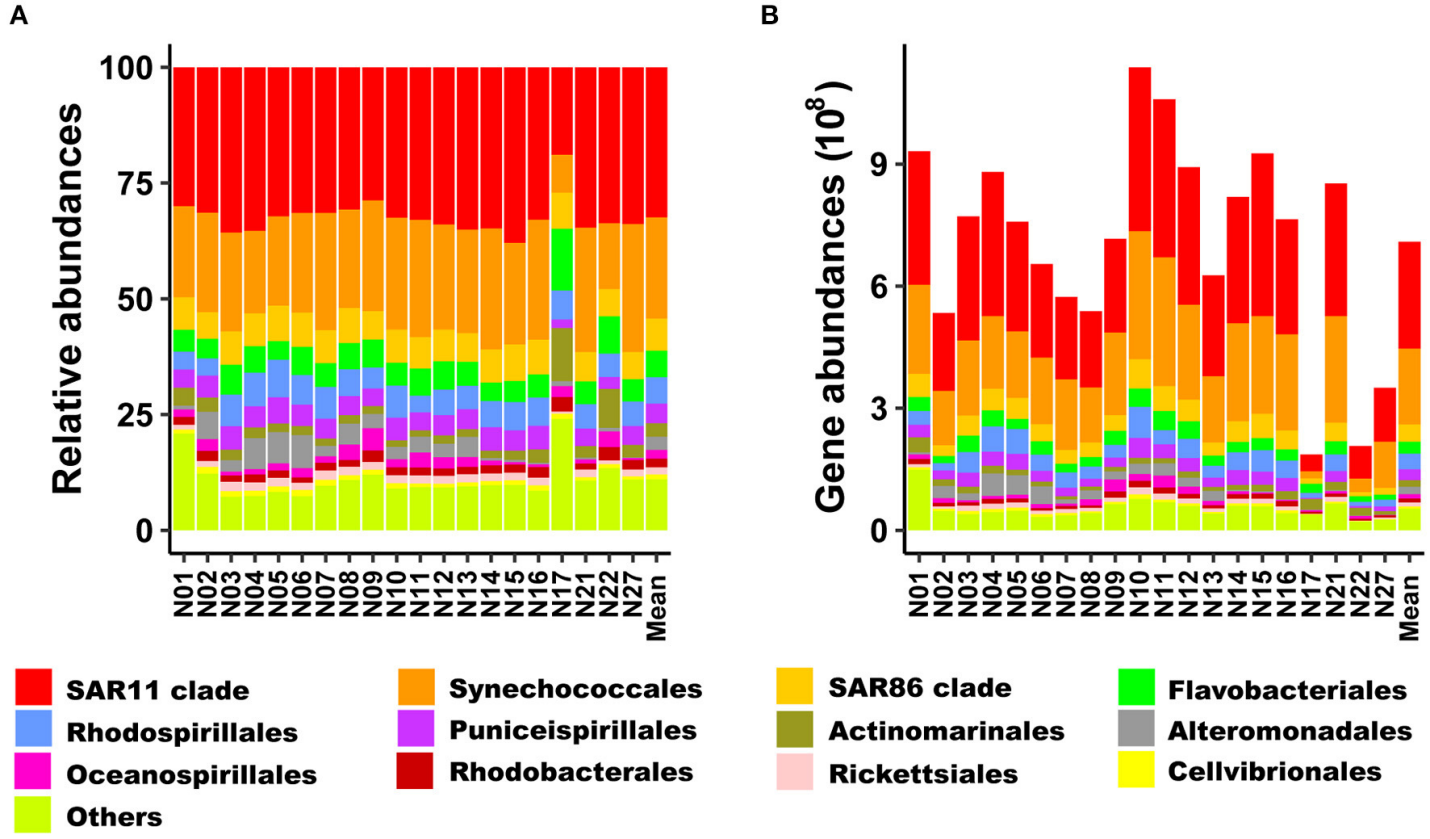
Bacterial community composition of relative abundance and rRNA gene abundance at the order levels **(A,B)**, respectively. Only the most abundant orders (12) are shown. The other orders are presented as “Others”.

Strong linear relationships (*R*^2^ > 0.988) were found between the added copy numbers of spike-in standards and the abundances of spike-in OTU sequences recovered from sequencing for all samples (Supplementary Table 2). The quantitative taxonomic abundances calculated from spike-in standards averaged 7.09 × 10^8^ copies L^−1^ (range: 1.86 × 10^8^-1.14 × 10^9^ copies L^−1^) for bacterial communities (Figure 3B). At the phylum level, both relative and quantitative sequence abundances derived from all samples were dominated by Proteobacteria (average about 65.4% and 4.6 × 10^8^ copies L^−1^, respectively), followed by Cyanobacteria (average about 22.0% and 1.9 × 10^8^ copies L^−1^, respectively). These two phyla accounted for more than 87% of the total relative abundance. The α-proteobacteria and γ-proteobacteria dominated within Proteobacteria, accounting for 48.7 and 15.3% of total sequences, respectively (Supplementary Figure 3).

Bacteroidetes and Actinobacteria were ubiquitous in the investigated region and present in high relative abundance at some stations (e.g., stations N17 and N22, Supplementary Figure 3A). At the order level, the SAR11 clade within the Proteobacteria dominated in both relative (average of 32.4%) and quantitative (4.1 × 10^7^−4.0 × 10^8^ copies L^−1^ with an average of 2.6 × 10^8^ copies L^−1^) sequence abundances at all stations and sequences assigned to Synechococcales (Cyanobacteria) accounted for 21.9% of total sequences (Figure 3).

**Figure 4:**
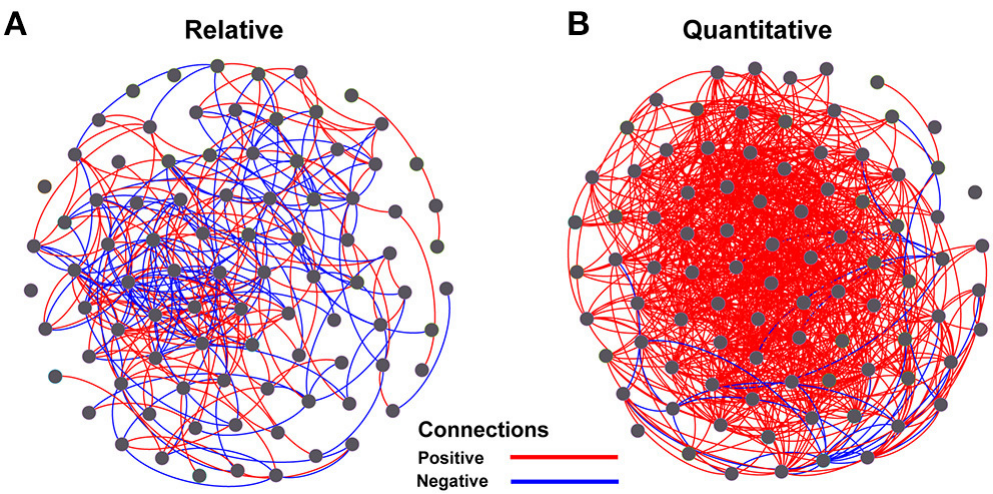
Network analysis of relationships among OTUs for relative abundance **(A)** and quantitative abundance **(B)** of bacterial communities. The OTUs with relative abundances >0.1% of total sequences were included to calculate pairwise Spearman correlations using the function corr.test() in the package “psych,” with *p*-values adjusted using the “fdr” method. Only significant correlations (adjusted *p* < 0.05 and *r* ≥ 0.6) were used to construct the network using the software Gephi. Red and blue lines represent significant positive and negative correlations, respectively.

**Figure 5:**
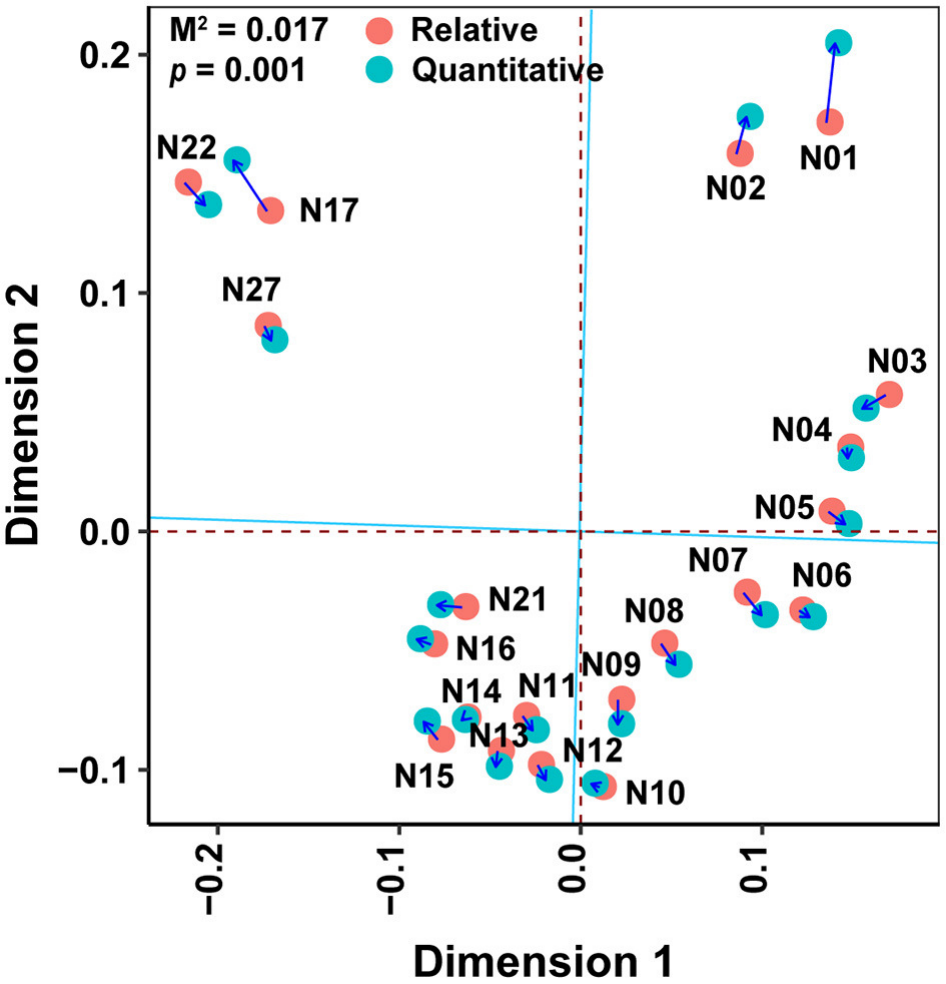
Procrustes analysis of relative abundance against quantitative abundance in presenting bacterial distribution patterns obtained from Principal Coordinates Analysis. Principal Coordinates Analysis based on Bray–Curtis distance of bacterial communities. The M^2^ that was approximated to zero indicated the remarkable agreement of comparison. The statistical significance was measured by the Monte Carlo test.

The Spearman correlation coefficients between relative and quantitative abundances were <0.8 in 46.2% of the cases, and 7.3% of the Spearman correlation coefficients were not significant (adjusted *P* > 0.05; Supplementary Figure 4). Furthermore, the community connections revealed by relative and quantitative abundances were markedly different, and the latter showed more complex and tighter connections (Figure 4, Table 1). Although equal numbers of OTUs were included in the analyses, compared to relative abundances, the quantitative abundances were associated with: (i) twice as many connections, with much more positive (1005 vs. 151) and fewer negative (29 vs. 143) connections; (ii) a higher average degree of clustering and clustering coefficient; and (iii) shorter average path length and lower modularity (Table 1). Procrustes analysis showed remarkable agreement between relative and quantitative abundances in presenting the distribution patterns of bacterial communities with *M*^2^ = 0.017 and *p* = 0.001. Moreover, it was noteworthy that bacterial communities, based on both relative and quantitative abundances presented distinct geographical patterns with spatially adjacent stations clustered together (Figure 5).

**Table 1:** Network characteristics of bacterial community based on relative and quantitative abundances.

**Attributes**	**Relative**	**Quantitative**
Node	90	90
Average degree	6.5	23.0
Average clustering coefficient	0.49	0.63
Edge	294	1,034
Positive edge	151	1,005
Negative edge	143	29
Average path length	3.02	1.98
Modularity	11.45	0.26

*Pairwise Spearman correlation coefficients for OTUs with relative abundance >0.1% of total sequences were calculated using the function corr.test() in the package “psych.” The p-values were adjusted using the “fdr” method. Only significant correlations (adjusted p <0.05 and r ≥ 0.6) were used to analyze network attributes via the software Gephi*.

### Potentially Important Factors Influencing the Bacterial Communities

**Figure 6:**
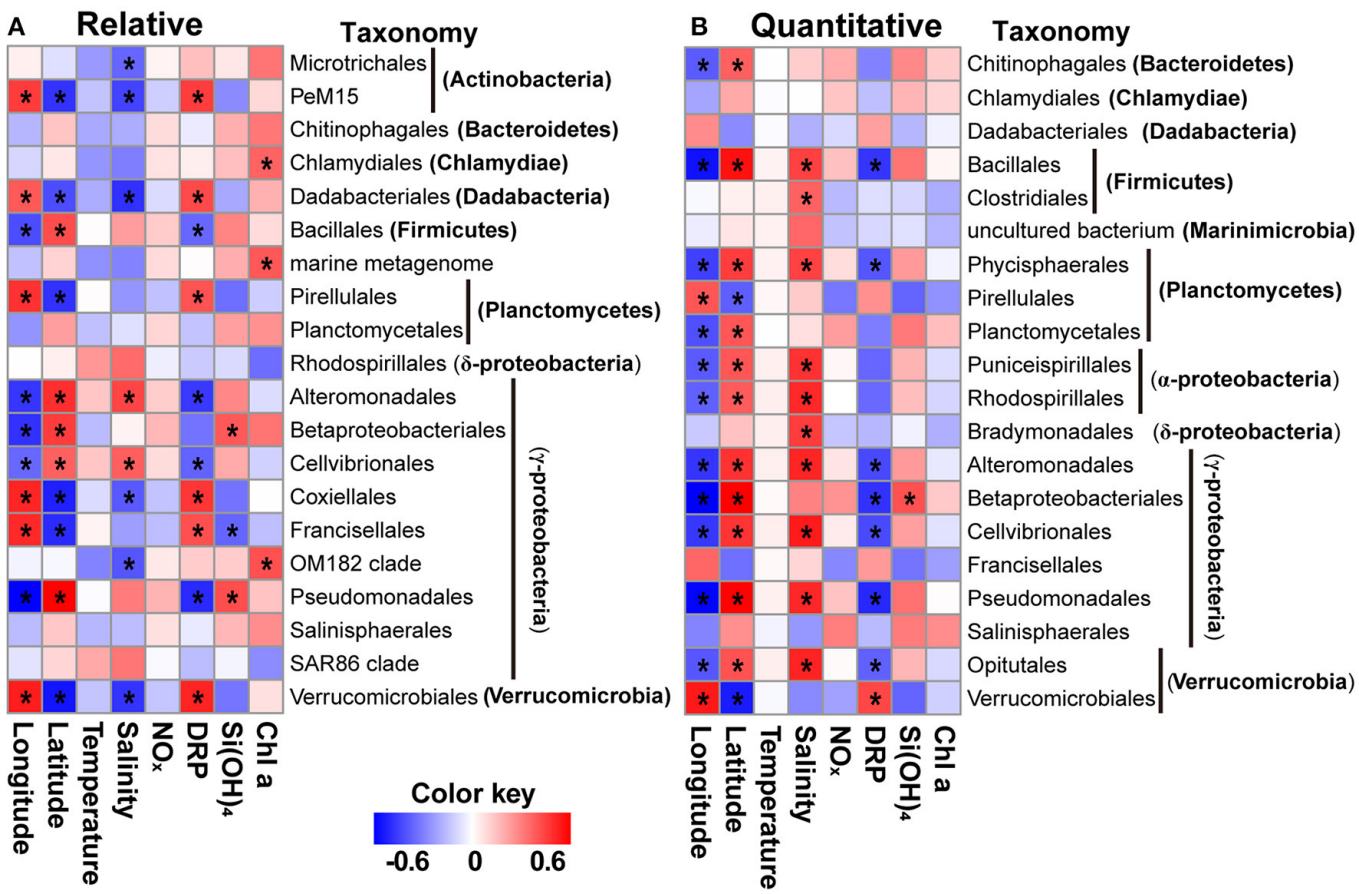
Heatmaps showing significant correlations between specific orders **(A,B)** of bacteria and environmental variables (e.g., temperature, salinity, nutrients, and Chl *a*) and geographic factors for relative and quantitative abundances using a sparse partial least squares approach. The orders present in all samples were used. Significant correlations (|*r*| > 0.5) are labeled with asterisks. NO_x_, nitrite + nitrate; DRP, dissolved reactive phosphorus.

No significant relationship with measured environmental and geographic variables was found for alpha diversity indices or quantified rRNA gene abundances, except for bacterial abundances and NO_x_ concentrations, which were positively correlated with Shannon and PD indices, respectively (Figure 2A). The effects of environmental and geographic factors on community composition were evaluated using Mantel tests (Table 2), VPA (Supplementary Figure 5), and sPLS analyses (Figure 6, Supplementary Figure 6).

**Table 2:** Mantel tests for the correlations between environmental variables, geographic factors, and bacterial composition based on Bray-Curtis distance for relative and quantitative abundances.

	**Relative**		**Quantitative**	
	***r***	***p***	***r***	***p***
Temperature	0.054	0.283	0.034	0.346
Salinity	**0.396**	**<0.001**	**0.429**	**<0.001**
NO_x_	0.066	0.273	0.106	0.235
DRP	**0.294**	**0.002**	**0.322**	**0.001**
Si(OH)_4_	**0.353**	**0.025**	**0.330**	**0.025**
Chl *a*	**0.367**	**0.014**	**0.291**	**0.035**
Bacteria	−0.102	0.719	−0.063	0.649
Envdist	**0.451**	**0.002**	**0.463**	**0.001**
Geodist	**0.738**	**<0.001**	**0.785**	**<0.001**

*Significance tests were set at 9,999 permutations and p-values (<0.05) are in bold. NO_x_, Nitrite + Nitrate; DRP, dissolved reactive phosphorus; BP, bacterial production; Bacteria, bacterial abundance; Envdist, pairwise Euclidean distances of environmental factors; Geodist, pairwise geographical distances based on Cartesian coordinates generated from longitude and latitude. Environmental variables were scaled to zero mean and unit variance*.

The variations of whole bacterial communities based on the Bray-Curtis distance were significantly correlated with salinity (*r* = 0.396, *P* < 0.001 and *r* = 0.429, *P* < 0.001 for relative and quantitative abundances, respectively), DRP concentrations (*r* = 0.294, *P* = 0.002 and *r* = 0.322, *P* = 0.001 for relative and quantitative abundances, respectively), Si(OH)_4_ concentrations (*r* = 0.353, *P* = 0.025 and *r* = 0.330, *P* = 0.025 for relative and quantitative abundances, respectively), Chl *a* concentrations (*r* = 0.367, *P* = 0.014 and *r* = 0.291, *P* = 0.035 for relative and quantitative abundances, respectively), and geographic distances (*r* = 0.738, *P* < 0.001 and *r* = 0.785, *P* < 0.001 for relative and quantitative abundances, respectively) (Table 2). Furthermore, the VPA showed that purely geographic factors might play significant roles in shaping bacterial communities based on both relative (30.2%) and quantitative (31.9%) abundances, but purely measured environmental factors explained no significant percentage of community variance. A large percentage of community variance was unexplained based on relative (47.8%) and quantitative (47.0%) abundances (Supplementary Figure 5).

We used sPLS to identify and visualize significant correlations between subgroups of bacteria and each environmental parameter and geographic factor, and in this way, we were able to reveal many significant relationships (Figure 6, Supplementary Figure 6). In terms of both relative and quantitative abundances, groups such as Dadabacteria and Gammaproteobacteria were correlated with longitude and latitude, and bacteria recovered as phyla (Dadabacteria and Firmicutes) were correlated with DRP concentrations (Supplementary Figure 6). However, the relative or quantitative abundance of no phylum was found to be correlated with temperature, NO_x_, or Chl *a* concentrations (Supplementary Figure 6). There were similar patterns at the order level for both relative and quantitative abundances. For example, the abundance of groups such as Alteromonadales, Bacillales, Betaproteobacteriales, cellvibrionales, Pirellulales, Pseudomonadales, and Verrucomicrobiales was correlated with longitude and latitude, whereas the abundances of lineages were uncorrelated with temperature and NO_x_ concentrations (Figure 6). Discrepancies of correlations with geographic factors, nutrients, and Chl *a* were also notably apparent for the relative and quantitative abundances of bacterial subgroups. For example, geographic factors correlated with the relative abundances of groups such as Ianctomycetes, Margulisbacteria, and Verrucomicrobia, but not for their quantitative abundances, whereas only the quantitative abundances of the orders Chitinophagales, Phycisphaerales, Planctomycetales, Puniceispirillales, and Opitutales were correlated with geographic variables (Figure 6, Supplementary Figure 6).

## Discussion

### Bacterial Community Composition Across the Subtropical NWPO

Although HTQS does not preclude a PCR-bias effect, which would impact absolute microbial abundances, it could, to some extent, enhance the significance of HTS-based microbiome studies (Tourlousse et al., 2017; Wang et al., 2018; Yang et al., 2018; Jiang et al., 2019; Mou et al., 2020). In our study, the strong linear relationships observed between the added copy numbers of spike-in standards and the abundances of spike-in OTU sequences (Supplementary Table 2) suggested that HTQS might be a robust method for quantifying the sequence abundances. Our observations of total bacterial 16S rRNA abundances were within the range of bacterial 16S rRNA abundances (1.78 × 10^8^−5.4 × 10^9^ copies L^−1^) reported in the surface seawater of the western North Atlantic, based on the HTQS method (Wang et al., 2018) and consistent with bacterial 16S rRNA gene abundances based on real-time PCR in the surface seawater of the South Pacific Gyre (5.96 × 10^8^−2.55 × 10^9^ copies L^−1^) (Yin et al., 2013). Quantitative abundances of the SAR11 16S rRNA gene in our samples were also in accordance with previous observations. For example, a mean quantitative abundance of 2.6 × 10^8^ copies L^−1^ has been reported for SAR11 in surface seawater of the western North Atlantic based on the HTQS method (Wang et al., 2018), and SAR11 gene abundances estimated by quantitative PCR (qPCR) have been reported to fall in the range 0.3 × 10^8^−6.3 × 10^8^ copies L^−1^ at Station ALOHA (Eiler et al., 2009).

Our results were also consistent with the SAR11 cell abundances of 2 × 10^8^ cells L^−1^ estimated from FISH in the Sargasso Sea (Morris et al., 2002), if there is one gene copy number per SAR11 cell (Giovannoni et al., 2005). It should be noted, however, that there was no correlation between bacterial abundance enumerated with flow cytometry and 16S rRNA gene abundance based on the HTQS method (Figure 2A). Because there are significant methodological differences between HTQS and flow cytometry, it was reasonable to anticipate that they do not provide estimates of the same characteristics of bacterial communities.

At the basin scale, the bacterial communities were dominated at the phylum level by Proteobacteria, followed by Cyanobacteria (Supplementary Figure 3). Furthermore, the SAR11 (Proteobacteria) and Synechococcales (Cyanobacteria) lineages were the predominant groups at the order level in our study (Figure 3).

With some exceptions, our results were generally consistent with the results of previous studies in oligotrophic surface seawater at both the global and local scales (Yin et al., 2013; Sunagawa et al., 2015; Lindh, 2017; Shulse et al., 2017; Li et al., 2018). Based on metagenomic data, for example, Sunagawa et al. (2015) found that typical members of the Proteobacteria (i.e., SAR11 and SAR86 clades) and Cyanobacteria were the dominant bacteria in samples collected across all oceanic provinces during the Tara Oceans expedition. Moreover, Proteobacteria and Cyanobacteria have generally been shown to be the predominant phyla in surface seawater based on either 16S rRNA gene clone libraries from the center to the edge of the South Pacific Gyre (Yin et al., 2013) or HTS results of the 16S rRNA gene (V3 region) in the NWPO (Li et al., 2018). However, Lindh (2017) found that Cyanobacteria dominated bacterial communities along a transect from Honolulu to Station ALOHA based on HTS of the V3–V4 region of the bacterial 16S rRNA gene, and Li et al. (2018) found that Bacteroidetes was the second most abundant phylum at several stations in the northwestern Pacific. These discrepancies could be partially attributed to the different methodologies used, such as PCR primers. Previous studies, for example, have revealed that different primer sets can result in biased diversity metrics for bacterial communities (Cai et al., 2013). The fact that the primers used by Lindh (2017) and by us were the same, but that there were differences in the community compositions based on those primers indicates that factors other than primer sets contributed to the observed differences. Li et al. (2018) have shown that either Cyanobacteria or Proteobacteria can be the dominant bacterial phylum in the surface water of the NWPO. We, therefore, hypothesize that differences of the environmental factors associated with currents, water masses (Tseng et al., 2016), and/or physical processes such as eddies and upwelling might play a more important role than methodological differences (i.e., different primers) in explaining the differences of bacterial community composition in the different studies.

### Potentially Important Factors That Influence Bacterial Communities

We identified environmental variables (i.e., salinity, nutrient, and Chl *a* concentrations) and geographic factors as potential determinants of bacterial communities (Table 2, Supplementary Figure 5). Moreover, these potential determinants were correlated to varying degrees with different bacterial lineages (Figure 6, Supplementary Figure 6). More specifically, Chl *a* concentrations were found to be potentially important factors that shaped whole bacterial communities (Table 2), but they were uncorrelated with specific lineages at the phylum level (Supplementary Figure 5). Similarly, Li et al. (2018) found that bacterial community structure is positively correlated only with temperature, whereas, at the phylum level, Proteobacteria, and Cyanobacteria are weakly correlated with both temperature and nutrients, and Chloroflexi are negatively correlated with ammonium concentrations in the surface water of the NWPO.

Partial least squares regression analyses revealed that latitude, longitude, and temperature are associated with specific groups of prokaryotic taxa in the western North Atlantic (Wang et al., 2018). In addition, Lindh et al. (2018) have shown that the effects of environmental and spatial factors vary in terms of their ability to explain whole bacterioplankton assemblages and subgroups thereof in the Clarion-Clippperton zone of the Pacific Ocean and along global ocean transects (i.e., the TARA and Malaspina expeditions). The implication is that the mechanisms that shape bacterioplankton communities differ between whole communities and subgroups. To comprehensively understand the biogeography of marine bacteria and the mechanisms that structure marine bacterial communities, the distinction between different taxonomic levels should be taken into consideration in future studies.

Our results revealed that spatial factors might play more important roles than environmental variables in regulating bacterial communities (Table 2, Supplementary Figure 5). Mantel tests revealed that spatial factors were more significantly correlated with bacterial communities than environmental variables (Table 2), and VPA showed that purely spatial factors could explain a large percentage of the variance of bacterial community composition, whereas the environmental metrics that we measured explained no significant percentage of the variance (Supplementary Figure 5). By contrast, Lindh et al. (2018) found that purely environmental conditions accounted for a significant percentage of the variance of epipelagic (5, 80, and 125 m) bacterioplankton composition in the Clarion-Clipperton Zone of the Eastern North Pacific. Based on rRNA gene data from the Tara oceans, the same study observed that environmental conditions might play a more important role than spatial differences in structuring epipelagic (0–200 m) bacterial assemblages. Water temperature was recognized to be the most important driver of the selection of surface prokaryotes sampled during the Malaspina and TARA Oceans expeditions (Logares et al., 2020). One possible explanation for these disparate results is that environmental heterogeneity was much higher in previous studies than in this study. For example, water temperatures ranged from 28.55 to 29.68°C in this study, but it fluctuated between 12.79 and 27.63°C in the epipelagic waters of the Clarion-Clipperton Zone during the study of Lindh et al. (2018) and from 15.7 to 29.3°C across the “Meta-119 Malaspina dataset” during the study of Logares et al. (2020).

Our results also revealed that environmental variables such as salinity, DRP, and Si(OH)_4_ concentrations were correlated with spatial factors (Figure 2A), and that correlation could explain their shared effect on the variations of bacterial community structure (Supplementary Figure 4). It should be noted that there was a large percentage of community variance that was unexplained by the spatial and environmental variables in our study (Supplementary Figure 5). We speculate that this unexplained community variance might have been due to unmeasured but important environmental factors (e.g., wind speeds and water current velocities), ecological processes (e.g., dispersal and ecological drift), and/or biological variables, such as taxon-specific mortality by grazers, viral infection, and biotic interactions.

### Comparing the Quantitative and Relative Abundances of Members of the Bacterial Communities

We evaluated community structure, distribution patterns, and the potentially important determinants of bacterial community composition in terms of both relative and quantitative abundances. The distribution patterns of the relative and quantitative abundances of whole communities of bacteria were similar (Figure 5) and were affected in similar ways by potential determinants of community structure (Table 2, Supplementary Figure 5). At the subgroup level, the distributions and potential determinants of relative and quantitative abundance differed (Table 1, Figures 4, 6, Supplementary Figures 4, 6). The attributes of networks constructed from relative and quantitative abundances were quite different (Figure 4, Table 1). Because positive connections might tend to destabilize bacterial communities, or vice versa (de Vries et al., 2018), our results may indicate that bacterial networks appear to be less stable when they are assessed in terms of quantitative abundance vs. relative abundance. Furthermore, the Spearman correlation coefficients between relative and quantitative abundances were <0.8 in 46.2% of cases, and 7.3% were not significant correlations (adjusted *P* > 0.05; Supplementary Figure 4). Hence there were significant differences between relative and quantitative abundances at the subgroup level.

By combining the HTS approach with single-cell enumeration technology, Props et al. (2017) showed that there is no inevitable correlation between enrichment (increase in relative abundance) and outgrowth (increase in absolute abundance) of taxa. This result highlights the need to consider both relative and absolute abundances to present a comprehensive interpretation of ecological scenarios. The consideration of the potentially important factors that might shape bacterial community composition in this study was based on relative and quantitative abundances that revealed similar patterns for whole communities, but the patterns were dissimilar for specific subgroups (Table 2, Figure 6, Supplementary Figures 4, 6). Previous studies have shown significantly different trends in the abundances of major groups within microbial communities, based on relative abundances generated using HTS and measured abundances estimated by direct or indirect measurements with adenosine triphosphate, flow cytometry, quantitative real-time PCR (qPCR), concentrations of phospholipid fatty acids or microbial carbon biomass (Zhang et al., 2017), and internal standard strains (Yang et al., 2018). In addition, Vandeputte et al. (2017) revealed that quantitative microbiome profiling plays a very important role in the analysis of relationships between species that occur together and the characterization of changes of pathogenic microorganisms through parallelization of HTS and flow cytometric enumeration technology. Collectively, the trends of the relative and quantitative abundances of marine bacteria might be significantly different at subgroup levels. A consideration of the quantitative and relative abundances of members of the bacterial community might therefore be pivotal in revealing important aspects of marine microbial ecology.

## Conclusions

Although bacteria play a pivotal role in shaping ecosystems and contributing to the cycling of elements and flow of energy in the oceans, few studies have addressed the basin-scale distribution of marine bacteria based on the quantification of bacterial abundance. This study revealed that total 16S rRNA gene copies ranged from 1.86 × 10^8^ to 1.14 × 10^9^ copies L^−1^ in the subtropical NWPO. The spatial distributions of the bacterial communities were distinct, and geographic factors appeared to play important roles in structuring bacterial communities. Our analyses indicated that consideration of both the relative and quantitative abundances of bacteria in a community might help to reveal important aspects of marine microbial ecology. However, because our conclusions are based on analysis of only surface seawater sampled from the subtropical NWPO, further investigations are needed to extend this approach to subsurface waters (e.g., the deep chlorophyll maximum layer, twilight zone, and deep ocean), and/or to regions with gradients of environmental conditions (e.g., salinity and nutrient concentrations). Such studies would greatly enhance our knowledge of how ocean ecosystems work and how they may respond to climate change.

## Data Availability Statement

The datasets presented in this study can be found in online repositories. The names of the repository/repositories and accession number(s) can be found at: https://www.ncbi.nlm.nih.gov/, PRJNA614577.

## Author Contributions

BH and JK conceived and designed the study. JK and LW collected the samples. JK and JG conducted the experiments. JK analyzed the data. All authors wrote the manuscript.

## Conflict of Interest

The authors declare that the research was conducted in the absence of any commercial or financial relationships that could be construed as a potential conflict of interest.
