# Supplementary figures and images for: Patterns of Relative and Quantitative Abundances of Marine Bacteria in Surface Waters of the Subtropical Northwest Pacific Ocean Estimated With High-Throughput Quantification Sequencing
Source: Front Microbiol. 2021 Jan 21;11:599614. doi: 10.3389/fmicb.2020.599614 (PMC7859494; doi:10.3389/fmicb.2020.599614)

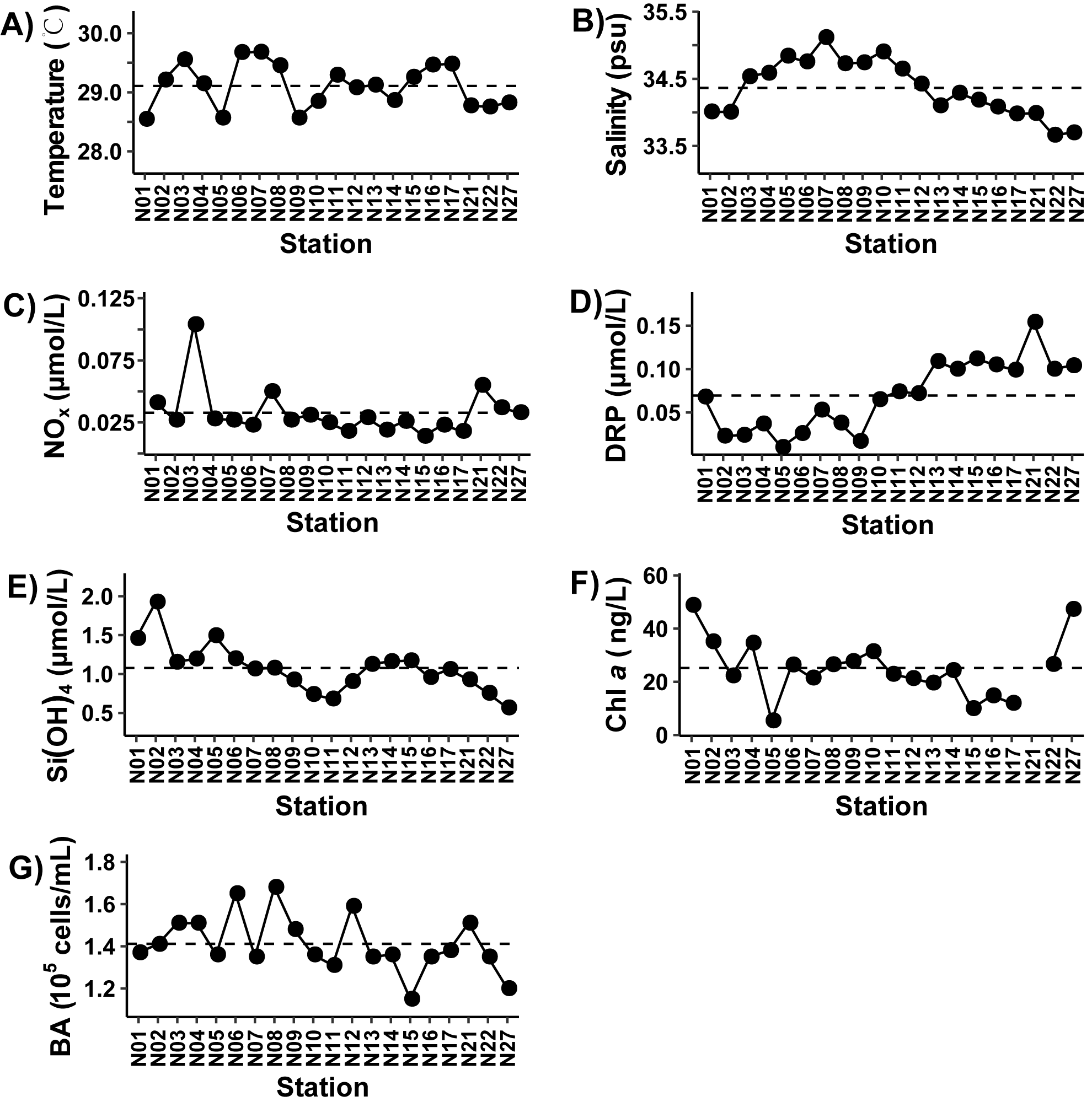

Supplement: Supplementary file 1 [file Image_1.TIF]

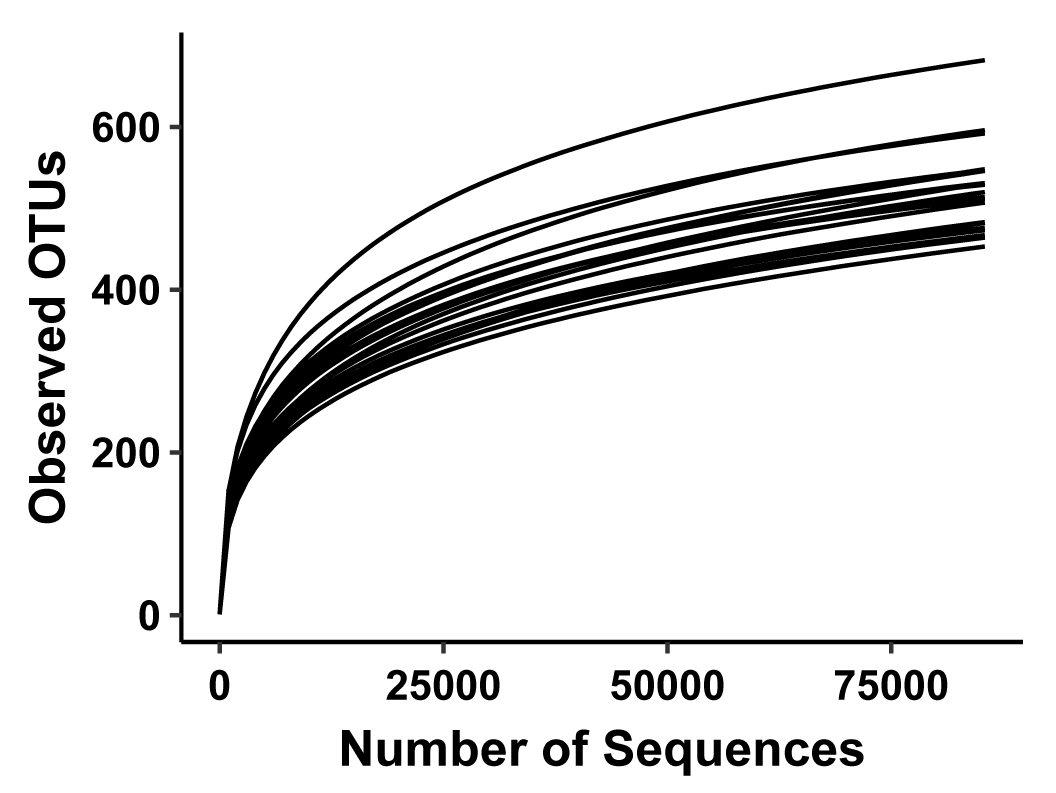

Supplement: Supplementary file 2 [file Image_2.TIF]

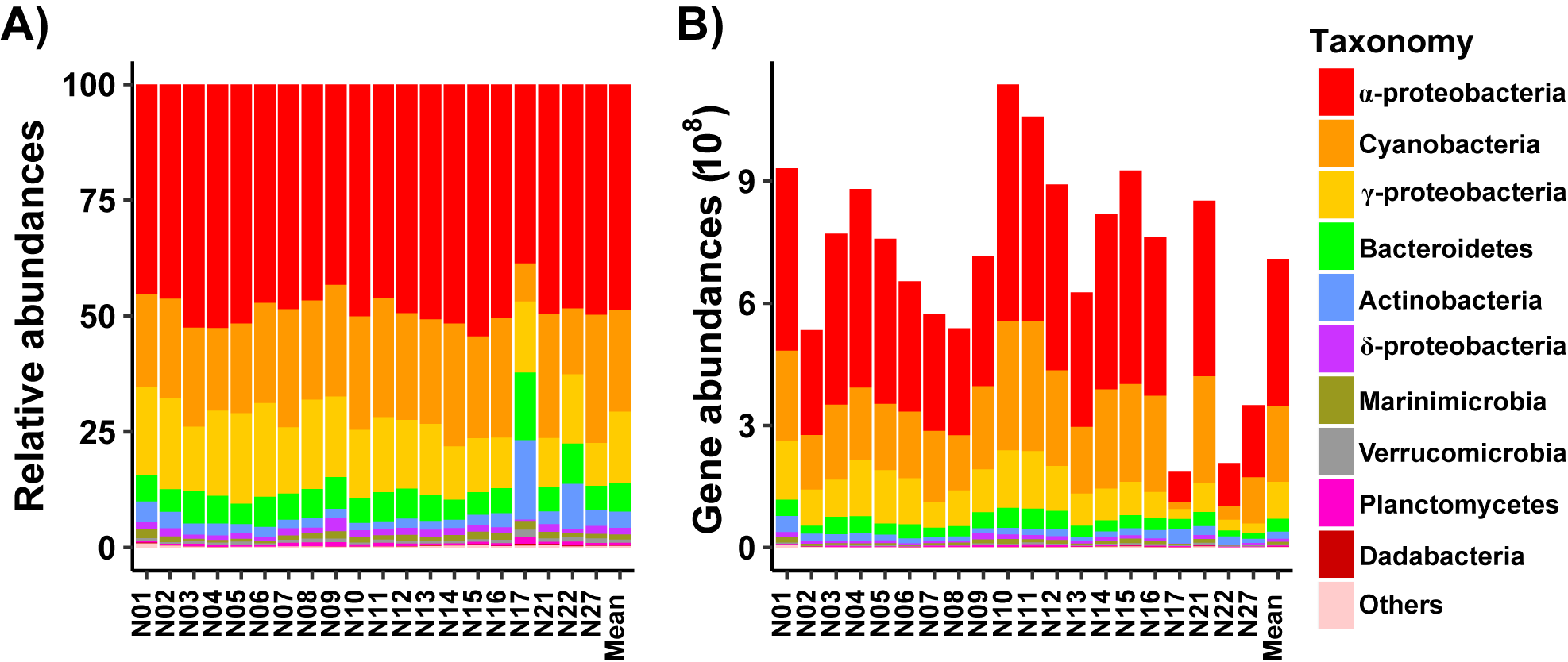

Supplement: Supplementary file 3 [file Image_3.TIF]

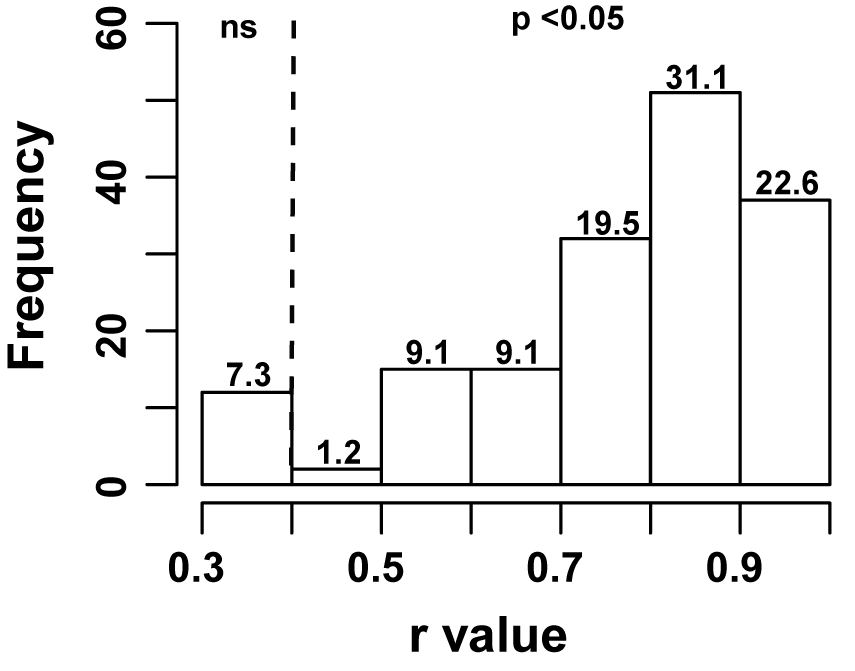

Supplement: Supplementary file 4 [file Image_4.TIF]

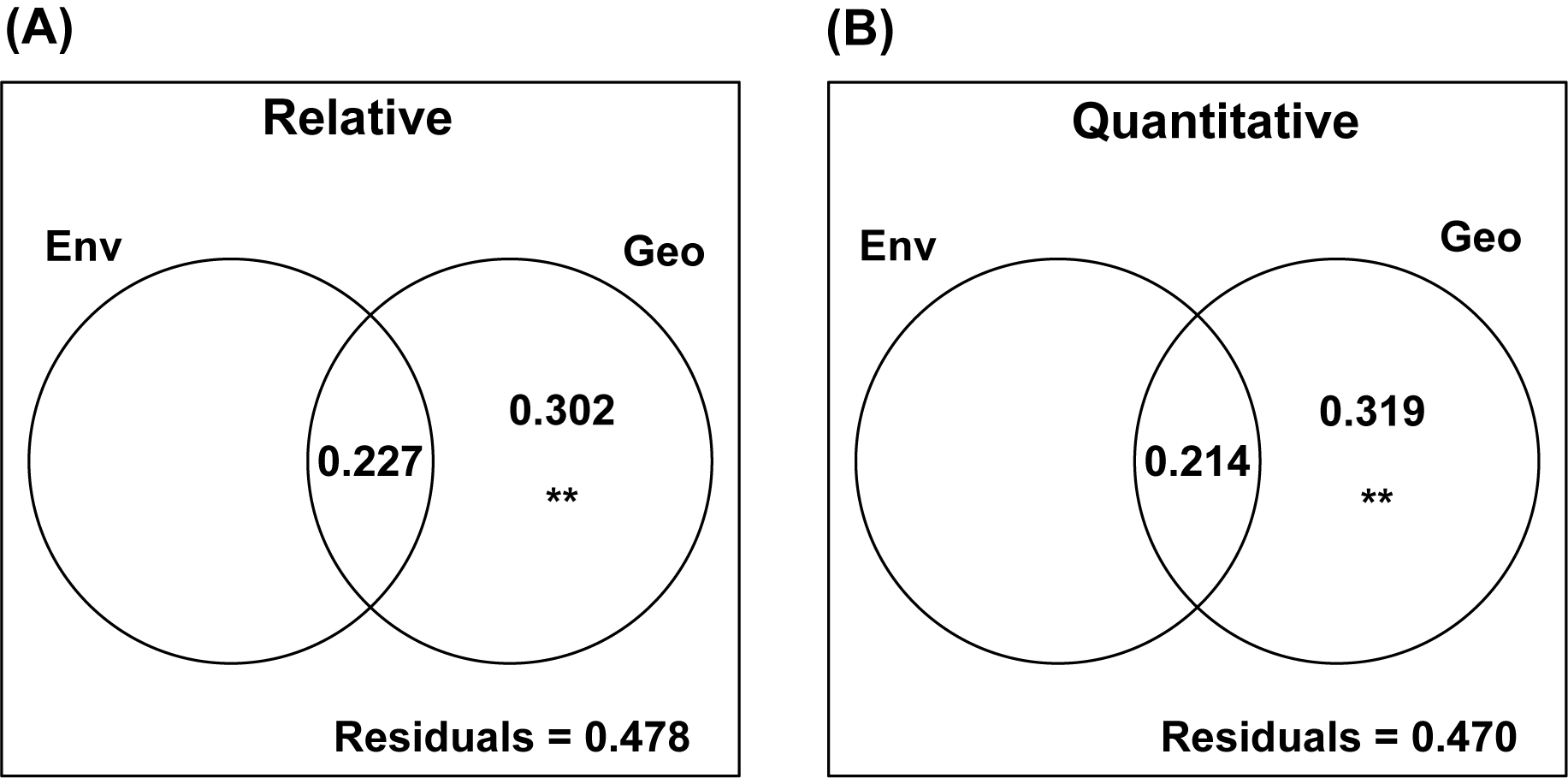

Supplement: Supplementary file 5 [file Image_5.TIF]

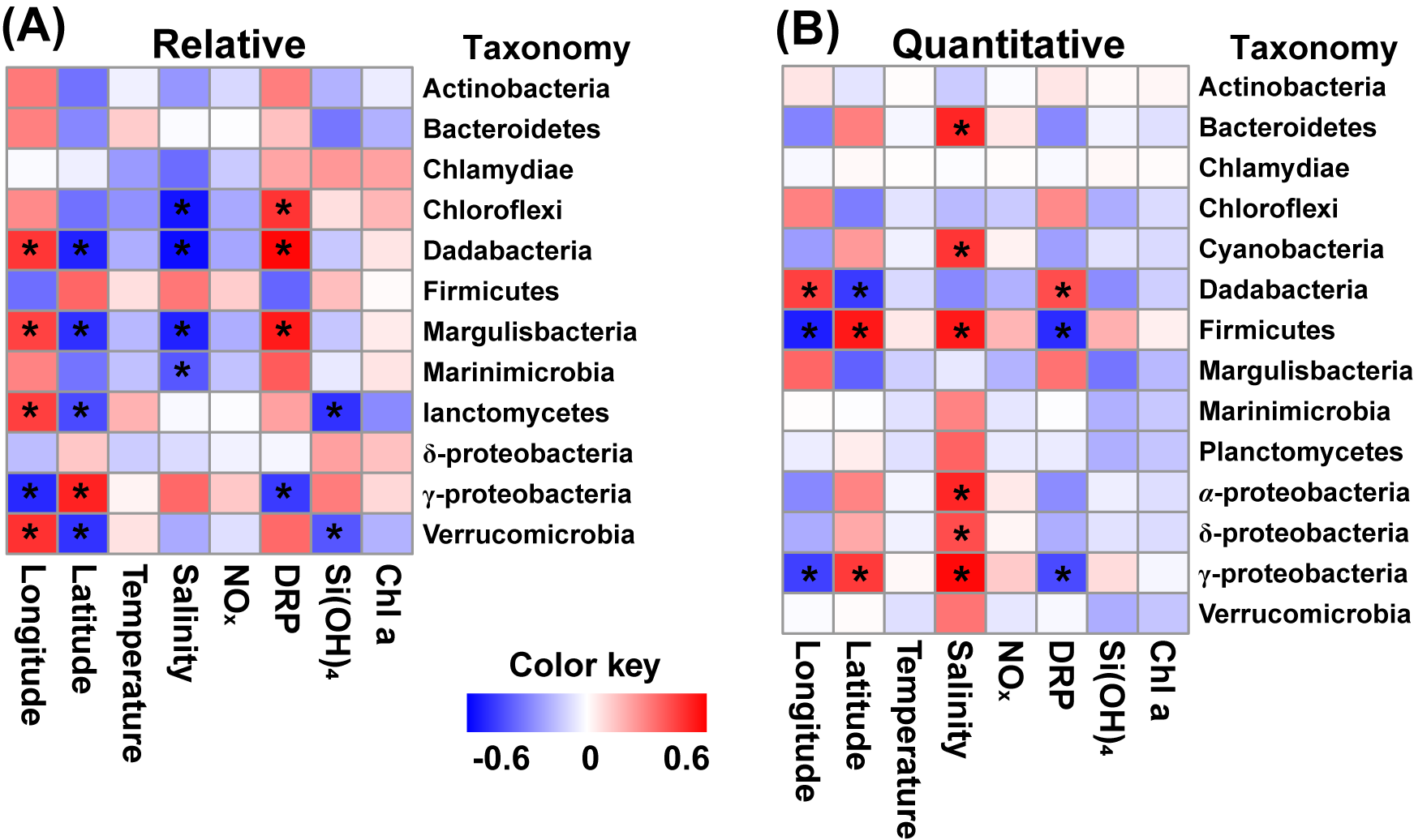

Supplement: Supplementary file 6 [file Image_6.TIF]
