# Supplementary material for: Patterns of Relative and Quantitative Abundances of Marine Bacteria in Surface Waters of the Subtropical Northwest Pacific Ocean Estimated With High-Throughput Quantification Sequencing
Source: Front Microbiol. 2021 Jan 21;11:599614. doi: 10.3389/fmicb.2020.599614 (PMC7859494; doi:10.3389/fmicb.2020.599614)
Supplement: Supplementary file 7 [file Data_Sheet_1.docx]

**Table S1 |**Environmental factors for the 20 surface seawater samples in the Northwest Pacific Ocean. Name, sample names used in this study; Date, day/month/year; Salinity, daily mean surface seawater salinity; NO_x_, nitrite + nitrate; DRP, dissolved reactive phosphorus; BA, bacterial abundance.

| **Name** | **Date** | **Longitude** | **Latitude** | **Temperature ℃** | **Salinity psu** | **NO_x_ μmol/L** | **Si(OH)_4_ μmol/L** | **DRP μmol/L** | **Chl *a* ng/L** | **BA 10^5^ cells/mL** |
| --- | --- | --- | --- | --- | --- | --- | --- | --- | --- | --- |
| N01 | 16/07/2018 | 126.21 | 27.52 | 28.55 | 34.01 | 0.041 | 1.458 | 0.068 | 48.8 | 1.37 |
| N02 | 17/07/2018 | 130.03 | 28.96 | 29.21 | 34.01 | 0.027 | 1.927 | 0.023 | 35.1 | 1.41 |
| N03 | 18/07/2018 | 133.90 | 28.25 | 29.55 | 34.53 | 0.104 | 1.153 | 0.024 | 22.3 | 1.51 |
| N04 | 19/07/2018 | 137.92 | 27.38 | 29.15 | 34.59 | 0.028 | 1.195 | 0.037 | 34.6 | 1.51 |
| N05 | 20/07/2018 | 142.45 | 26.25 | 28.57 | 34.84 | 0.027 | 1.495 | 0.010 | 5.4 | 1.36 |
| N06 | 21/07/2018 | 146.49 | 25.19 | 29.68 | 34.75 | 0.023 | 1.198 | 0.026 | 26.4 | 1.65 |
| N07 | 22/07/2018 | 150.18 | 24.11 | 29.68 | 35.12 | 0.050 | 1.067 | 0.053 | 21.5 | 1.35 |
| N08 | 23/07/2018 | 154.30 | 22.78 | 29.45 | 34.73 | 0.027 | 1.077 | 0.038 | 26.5 | 1.68 |
| N09 | 26/07/2018 | 161.40 | 20.28 | 28.57 | 34.74 | 0.031 | 0.926 | 0.017 | 27.7 | 1.48 |
| N10 | 27/07/2018 | 165.52 | 19.15 | 28.85 | 34.90 | 0.025 | 0.739 | 0.065 | 31.4 | 1.36 |
| N11 | 28/07/2018 | 169.47 | 17.96 | 29.29 | 34.65 | 0.018 | 0.681 | 0.074 | 22.9 | 1.31 |
| N12 | 29/07/2018 | 173.20 | 16.75 | 29.08 | 34.42 | 0.029 | 0.905 | 0.072 | 21.3 | 1.59 |
| N13 | 30/07/2018 | 176.73 | 15.53 | 29.12 | 34.10 | 0.019 | 1.126 | 0.109 | 19.6 | 1.35 |
| N14 | 31/07/2018 | -179.73 | 14.24 | 28.86 | 34.29 | 0.026 | 1.162 | 0.100 | 24.3 | 1.36 |
| N15 | 01/08/2018 | -175.97 | 12.80 | 29.26 | 34.19 | 0.014 | 1.172 | 0.112 | 10.0 | 1.15 |
| N16 | 02/08/2018 | -172.80 | 11.51 | 29.46 | 34.08 | 0.023 | 0.958 | 0.105 | 14.8 | 1.35 |
| N17 | 03/08/2018 | -169.20 | 10.00 | 29.48 | 33.98 | 0.018 | 1.060 | 0.099 | 12.0 | 1.38 |
| N21 | 04/08/2018 | -165.67 | 10.00 | 28.77 | 33.99 | 0.055 | 0.928 | 0.154 | - | 1.51 |
| N22 | 04/08/2018 | -163.20 | 10.00 | 28.75 | 33.66 | 0.037 | 0.754 | 0.100 | 26.6 | 1.35 |
| N27 | 05/08/2018 | -158.77 | 9.31 | 28.82 | 33.70 | 0.033 | 0.566 | 0.104 | 47.3 | 1.20 |

**Table S2 |** Standard curve equation based on spike-ins for each sample. y is the log-transformed spike-in OTU sequences, and x represents log-transformed copies of spike-ins added to the sample.

| **Sample** | **linear equation** | ***R*^2^** |
| --- | --- | --- |
| N01 | y=0.8989x-1.7019 | 0.995452 |
| N02 | y=0.9079x-1.7905 | 0.991326 |
| N03 | y=0.9211x-1.884 | 0.988271 |
| N04 | y=0.9082x-1.8637 | 0.993504 |
| N05 | y=0.9293x-1.8654 | 0.992048 |
| N06 | y=0.9124x-1.7221 | 0.992404 |
| N07 | y=0.9036x-1.769 | 0.993954 |
| N08 | y=0.9091x-1.8346 | 0.994006 |
| N09 | y=0.9164x-1.8029 | 0.989978 |
| N10 | y=0.9261x-1.9324 | 0.994018 |
| N11 | y=0.9098x-1.7948 | 0.99507 |
| N12 | y=0.9202x-1.7969 | 0.995836 |
| N13 | y=0.9099x-1.8449 | 0.996302 |
| N14 | y=0.9292x-1.9485 | 0.993356 |
| N15 | y=0.8938x-1.7718 | 0.992579 |
| N16 | y=0.8958x-1.828 | 0.995336 |
| N17 | y=0.9018x-1.4355 | 0.997542 |
| N21 | y=0.9186x-1.7668 | 0.995045 |
| N22 | y=0.913x-1.6748 | 0.991727 |
| N27 | y=0.9081x-1.7683 | 0.995709 |

**Figure S1 |** Distribution of physical, chemical and biological parameters, including surface seawater temperature **(A)** and daily mean salinity **(B)**, concentrations of NO_x_ **(C)**, DRP **(D)**, Si(OH)_4_ **(E),** and Chl *a* **(F)**, and bacterial abundance (BA) **(G)**.

**Figure S2 |** Rarefaction curves of the individual bacterial samples. Operational taxonomic units (OTUs) were assigned based on 97 % similarity.

**Figure S3 |** Bacterial community composition of relative abundance and rRNA gene abundance at phylum level **(A), (B)**, respectively. Sequences assigned to the Proteobacteria phylum was shown at class level. Only the most abundant phyla (10) are shown. The other phyla are presented as ‘Others’.

**Figure S4 |** Histogram showing *r* value distribution of pairwise Spearman correlation coefficients between relative and quantitative abundances for OTUs. The 164 OTUs present in all samples were used. The q-values were corrected using the package ‘qvalue’. The value on top of each bar shows the percentage of total comparisons. Dashed line denotes the separation of *p*-values. ns means not significant.

**Figure S5 |** Variation partitioning analysis (VPA) for relative **(A)** and quantitative (B) abundances of bacterial community. VPA shows the effects of environmental and geographic variables on the composition of the bacterial community. Values indicating the fraction of bacterial community variation explained or unexplained by environmental and geographic variables. ANOVA permutation tests were applied to assess the significance of the pure effect for Env and Geo, respectively. Significance levels are as follows: Values <0 are not shown, *** *p* <0.001. Env, environmental variables; Geo, geographic variables.

**Figure S6 |** Heatmaps showing significant correlations between specific phyla **(A)**, **(B)** of bacteria and environmental variables (e.g., temperature, salinity, nutrients and Chl *a*) and geographic factors for relative and quantitative abundances using a sparse partial least squares approach. The phyla present in all samples were used. Sequences assigned to the Proteobacteria phylum was shown at class level. Significant correlations (|*r*| >0.5) are labeled with asterisks. NO_x_, nitrite + nitrate; DRP, dissolved reactive phosphorus.
